# Changes in the Optical Properties of Rubber Exposed to High-Pressure Hydrogen Using Pulsed Terahertz Waves

**DOI:** 10.3390/polym15234530

**Published:** 2023-11-25

**Authors:** Mun-Young Hwang, Hyun Chul Lee, Hyeok-Jae Yang, Dae-Hyun Han

**Affiliations:** 1 Multi-Material Research Center, Korea Automotive Technology Institute, 55 Jingoksandanjungang-ro, Gwangsan-gu, Gwangju 62465, Republic of Korea; myhwang@katech.re.kr (M.-Y.H.);; 2 Department of Carbon Convergence Engineering, Wonkwang University, 460 Iskander-ro, Iksan-si 54538, Republic of Korea

**Keywords:** sealing rubber, high-pressure hydrogen, terahertz waves, optical properties, non-destructive testing

## Abstract

In this study, we investigated how high-temperature, high-pressure hydrogen affects the optical properties of three kinds of sealing rubber (chloroprene rubber, ethylene propylene diene monomer, and acrylonitrile butadiene rubber) using pulsed terahertz waves. The optical properties of the rubber samples were analyzed before and after exposure to hydrogen (80 °C and 200 bar) for 72 h. The results showed that the terahertz waves had a shorter time delay and a lower signal intensity for all rubber types. The exposure response intensity, refractive index, and absorption rate also changed in the frequency domain. Raman and Fourier transform infrared spectroscopy were used for comparison, and a few peak shifts were observed. However, the Raman spectra had low signal quality, and the laser damaged the specimen. The study demonstrates that terahertz waves can be used as a non-contact non-destructive testing technique to evaluate the changes in sealing rubbers after hydrogen exposure.

## 1. Introduction

As concerns grow over greenhouse gas emissions, air pollution, and fossil fuel depletion, the need for and importance of hydrogen fuel cell vehicles as an eco-friendly transportation option are increasing. Hydrogen fuel cell vehicles must safely store and use high-pressure hydrogen gas, making the integrity of pressure vessels and connections crucial. Rubber materials, often in the form of O-rings, are widely used in connections to prevent the leakage of high-pressure hydrogen gas [1].

In a high-pressure environment, hydrogen gas can penetrate the relatively porous rubber material, leading to changes in its properties, a phenomenon known as hydrogen blister fracture [2]. In the case of sealing rubber materials, any degradation in their properties could result in catastrophic accidents. Notable incidents include the Hindenburg airship disaster in 1937, which was caused by hydrogen exposure [3], and the Challenger space shuttle explosion in 1986, which resulted from O-ring failure arising from inadequate consideration of the operating environment [4]. 

Here, we briefly review research into the changes in the properties of rubber materials exposed to high-pressure hydrogen. Jung et al. measured charging amount and diffusivity of the three types of rubbery polymers, and evaluated solubility and permeability [5]. Yamabe et al. evaluated the failure of ethylene propylene diene monomer (EPDM) rubber exposed to high-pressure hydrogen ranging from 10 to 70 MPa while varying the temperature from 30 to 100 °C [6]. They also applied the acoustic emission (AE) technique to detect internal crack formation, such as blisters [7]. Yamabe et al. also evaluated the tensile properties of acrylonitrile butadiene rubber (NBR) with the addition of carbon black and silica [1]. 

Fujiwara et al. observed an increase in hydrogenation as the pressure applied to the rubber material increased, along with a change in volume. They exposed rubber materials to 100 MPa of high-pressure hydrogen and used various analytical instruments, including NMR (Nuclear Magnetic Resonance), FTIR (Fourier transform infrared), and Raman spectroscopy, to assess the impact of high-pressure hydrogen on the chemical bonds of the rubber material [8,9]. However, they did not discover any new chemical structures. 

Hydrogen exposure analysis methods include thermal desorption spectroscopy [10], pressure composition isothermal (PCI) analysis [11], mass change analysis [12], hydrogen permeation testing [13], electron microscopy including the scanning electron microscopy (SEM) and transmission electron microscopy (TEM) methods [14], spectroscopic analysis (Raman and FTIR) [15], and tensile testing [14]. Suitable assessment standards for rubber materials in high-pressure hydrogen environments are provided in the CSA/ANSI CHMC 2:19-2019 standard “Test methods for evaluating material compatibility in compressed hydrogen applications—Polymers” (abbreviated as CSA/ANSI CHMC 2) [16]. However, existing hydrogen exposure analysis methods have the following limitations: (1) In most cases, they can quantify the amount of hydrogen exposure, but the prediction of property changes is difficult. (2) The results of Raman or FTIR spectroscopy can vary significantly depending on the site of measurement, limiting reproducibility. (3) Tensile testing can produce significantly different results depending on the test conditions. 

In contrast, terahertz waves can be used for the analysis of the structure and state of matter [17,18]; that is, by the interaction of terahertz waves at specific frequencies with a material. Crucially, hydrogen molecules exhibit various vibration modes (stretching, bending, double bond vibrations, and rotation), giving them characteristic intrinsic vibrational properties [19].

Terahertz waves can be used to measure the vibrational modes of hydrogen molecules, allowing for the analysis of characteristic parameters such as the dielectric constant spectrum, absorption coefficient, and refractive index. Dielectric constant spectra are widely employed in material characterization using terahertz waves [20]. For example, Chang et al. analyzed the degree of thermal aging of polypropylene (PP) in a nitrogen test environment using terahertz dielectric spectroscopy [21], and Lee et al. evaluated the degree of thermal aging of cellulose compression plates based on the refractive index results of terahertz waves [22]. 

Therefore, in this paper, we aimed to analyze and validate the changes in the properties of rubber materials exposed to high-pressure hydrogen using terahertz waves. We selected specific conditions for high-pressure hydrogen exposure, including the temperature, pressure, and time, as single parameters. By comparing the changes in optical time delay and intensity in the time domain, we examined the characteristics of hydrogen exposure. Additionally, we tested the feasibility of the analytical technique by comparing refractive index and absorption spectra. The results of analysis were compared and evaluated alongside Raman and FTIR spectroscopy to assess the validity and potential of the proposed method.

## 2. Materials and Methods

### 2.1. Test Materials

To safely use hydrogen gas in high-pressure vessels, the O-ring materials used in piping and connections must have the follow characteristics: (1) chemical stability, (2) leak prevention, (3) heat resistance, (4) pressure resistance, and (5) ozone resistance.

Rubber materials can absorb permeating hydrogen gas in a process known as hydrogen sorption [23,24]. Hydrogen sorption occurs because of the interaction between rubber materials and hydrogen molecules, where hydrogen is chemically absorbed or permeates into the material. Hydrogen molecules are very small (0.1 Å) and highly diffusive, which can lead to increased hydrogen sorption in rubber materials under high-temperature and high-pressure conditions. Therefore, it is essential to evaluate the property changes resulting from hydrogen sorption. In this study, we selected three representative rubber materials, NBR, EPDM, CR(Chloroprene rubber) [25], commonly used for sealing in a range of applications. 

NBR rubber is a type of thermosetting rubber characterized by its non-polar and amorphous properties, which are influenced by the presence of nitrile groups. NBR rubber exhibits excellent resistance to oils, solvents, and various chemicals, along with high wear resistance and chemical durability. EPDM rubber possesses excellent durability against heat, ozone, sunlight, and weathering, making it suitable for use in various environments. It exhibits high resistance to various chemical substances such as water, steam, alkalis, and acids, and maintains stable properties even at high and low temperatures. CR is a polymer of chloroprene (2-chlorobutadiene). Additionally, it has low gas permeability and strong adhesive properties. CR rubber exhibits excellent weather, flame, wear, chemical, corrosion, and ozone resistance compared to other rubber materials. 

**Figure 1:**
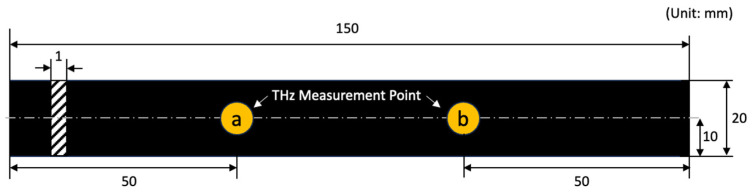
Specimen dimension and terahertz signal measurement points.

The sample size and measurement points are illustrated in Figure 1. We prepared three rubber specimens for each type, and measured terahertz signals at two measurement points per sample. The sample name was composed as “Rubber type_Sample No._Measurement point”.

### 2.2. High-Temperature and High-Pressure Hydrogen Gas Exposure Conditions

**Figure 2:**
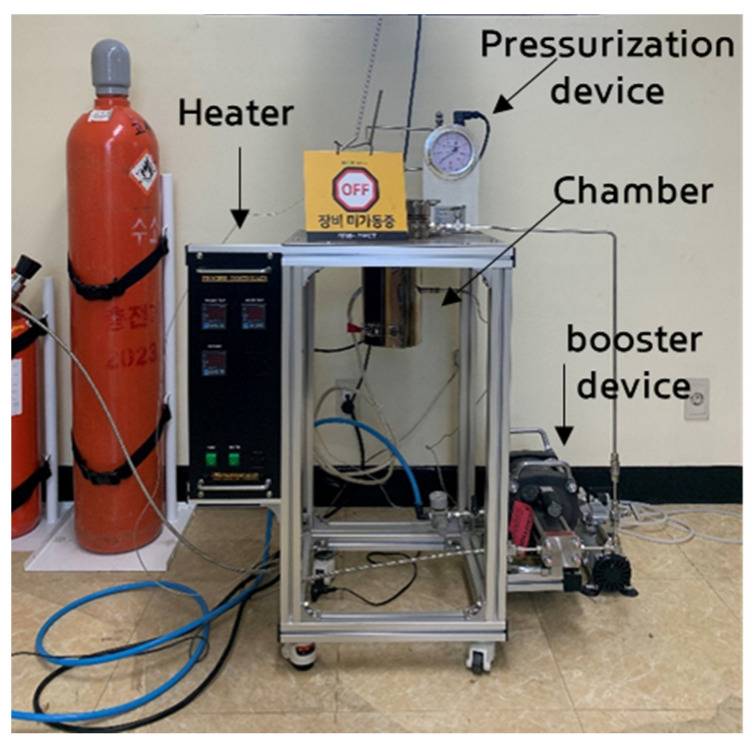
Experimental set-up for the hydrogen gas exposure test. (장비미가동중 means off.)

For the high-pressure hydrogen exposure loading tests on rubber specimens, a high-pressure hydrogen loading machine (Uto Engineering, Seoul, Republic of Korea) capable of reaching 25 MPa was used. The experimental set-up for the high-pressure hydrogen loading of rubber materials is shown in Figure 2. The device configuration consists of a heater for achieving high temperatures, booster device for producing high-pressure hydrogen, and pressurization device for increasing the pressure inside the chamber.

The process for removing impurities inside the high-pressure hydrogen tank is as follows: (1) the hydrogen storage tank is vacuumed to below atmospheric pressure, (2) hydrogen is added at a pressure of 20 MPa, and (3) hydrogen is released after filling is completed. This process was repeated twice to remove impurities inside the high-pressure hydrogen container. No separate decompression conditions were selected. The rubber specimens were dried for 48 h at 60 °C to minimize any effects of water exposure and volatile organic compound discharge.

High-temperatures activate hydrogen, which increases the amount of hydrogen penetration, whereas low temperatures reduce the penetration of the rubber with hydrogen. Thus, the specimens were kept at 80 °C, which is a harsh condition based on the material safety data sheet (MSDS) information for the three rubber samples.

The gas permeability of high-density polyethylene (HDPE) is much lower than those of NBR, CR, and EPDM. Generally, 13 h is sufficient for high-pressure hydrogen to penetrate the interior of HDPE in which hydrogen is not dispersed well and reach a balanced state [13]. Based on the reference [1] and experimental results, the longest exposure conditions were selected for which destruction by blisters would not occur after the hydrogen exposure test. Through monitoring of temperature changes, it was confirmed that there was no temperature change due to the hydrogen reaction of the material, so it was determined that an equilibrium state had been reached. Therefore, the high-pressure hydrogen exposure time was set to 72 h in this study.

### 2.3. Pulsed Terahertz Time-Domain Spectroscopy (Pulsed THz-TDS)

**Figure 3:**
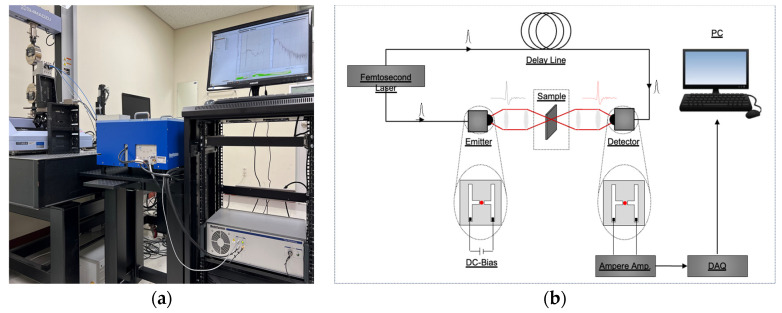
Test set-up for the pulsed THz-TDS experiments: (**a**) photograph and (**b**) schematic.

We employed a commercial fiber-coupled pulsed terahertz time-domain spectroscopy system (TERA K15, Menlo Systems Corp., Munich, Germany) to analyze the properties of the three rubber samples under the set conditions, as shown in Figure 3a. The primary components of the TERA K15 system are a femtosecond laser (T-Light, Menlo Systems Corp.), optical delay unit (ODU, Menlo Systems Corp.), photoconductive antenna (PCA) for the emitter (TERA 15-TX-FC, Menlo Systems Corp.), and detector (TERA 15-RX-FC, Menlo Systems Corp.

In summary, the THz-TDS system employs femtosecond laser-generated pump and probe beams to excite a terahertz radiation emitter, which generates terahertz pulses that are transmitted through the rubber samples, as shown in Figure 3b. The resulting sample signal, along with a reference signal generated by the terahertz detector, is processed and analyzed using signal processing techniques [17,18,26,27] to obtain spectra in the terahertz time and frequency domains.

### 2.4. Raman Spectroscopy

The Raman spectra of the rubber samples were acquired using an Xplora-Horiba spectrometer (Horiba Inc., Paris, France). The experiments were carried out at room temperature using an Olympus BX 40 microscope (Olympus, Tokyo, Japan) with a long-distance work objective and a magnification of ×50. A 1200 lines/mm grating was used to modify the simple spectrograph. A 785 nm laser light was used to illuminate the samples. The frequency range investigated was 400–3500 cm^−1^, with 50 accumulations and a 2 s acquisition period per spectrum. The lateral resolution was determined by the entrance slit (in our example, 100 mm), chosen objective, and grating. The spectral data were recorded using Horiba Labspec 6.

### 2.5. FTIR Spectroscopy

A Spectrum 100S (Perkin Elmer Co., Ltd., Shelton, CT, USA) was used to capture the FTIR spectra. The spectra were collected in transmission mode with a resolution of 4 cm^−1^ and 16 data collection iterations from 7800 to 450 cm^−1^. The diameter of the photodetector was about 8.94 mm. A metal plate measuring 50 by 95 by 2 mm with a 8 mm diameter hole served as the sample fixture.

## 3. Results

### 3.1. Terahertz Waveform in Time and Frequency Domain

**Figure 4:**
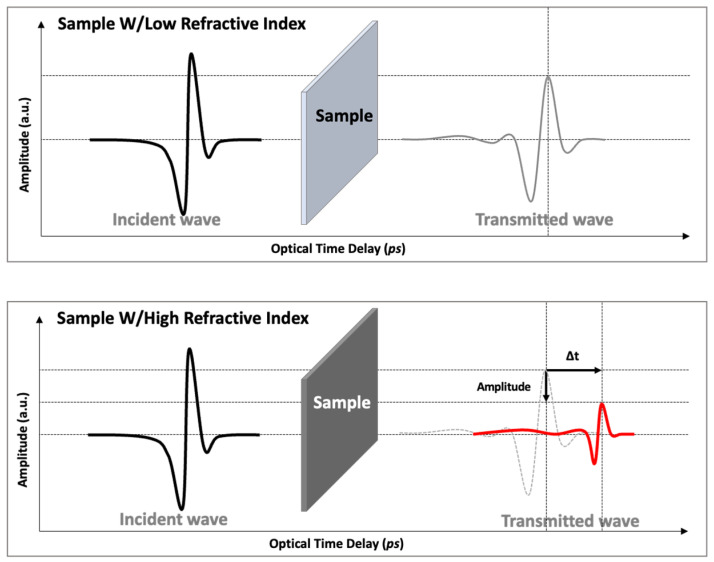
Illustration of the amplitude decrease of the pulsed terahertz wave and the optical time delay change as a result of differences in the refractive index.

When the rubber samples are placed into the beam path, the terahertz wave is influenced in two ways: (1) the incident terahertz wave will be absorbed or reflected according to the incident angle at the sample surface, and (2) the transmitted terahertz wave is delayed with respect to the air reference measurement because of the higher reflective index and, thus, longer optical path in the rubber samples, as illustrated in Figure 4. Analysis of the terahertz wave amplitude and position provides information on the refractive index and the absorption coefficient of the rubber samples by comparing the reference and acquired terahertz waves. Rubber samples after exposure to hydrogen have higher absorption coefficients and refractive indexes than those without, such that the amplitude of the transmitted terahertz wave will be smaller, and the peak of the terahertz wave will be detected later.

**Figure 5:**
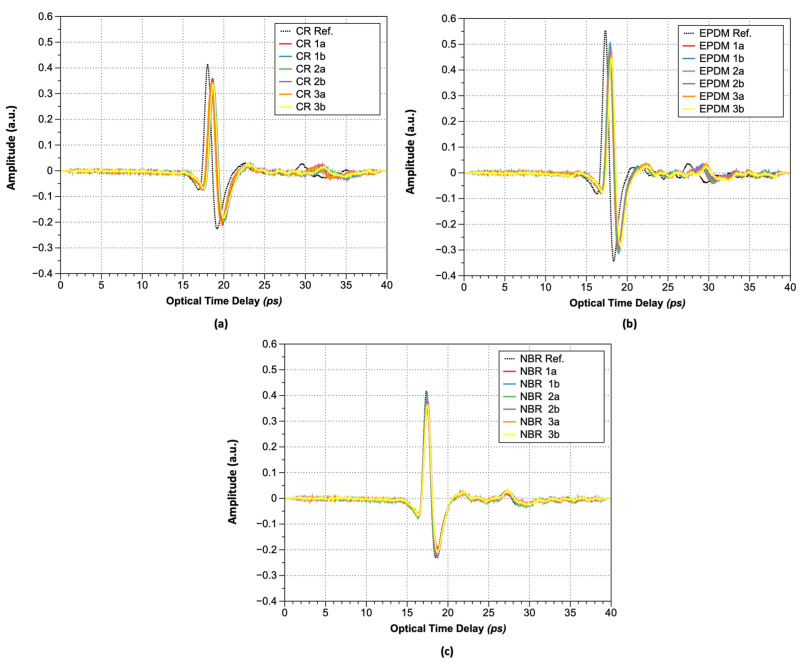
Transmitted pulsed terahertz wave: (**a**) CR; (**b**) EPDM; and (**c**) NBR.

Figure 5 shows the acquired terahertz waveforms after hydrogen exposure via THz-TDS. As shown, the amplitude of the maximum peak decreased from 0.42 to 0.34 arbitrary units (a.u.). The optical time delay value (∆*t*) corresponding to the maximum peak value also decreased from 18.00 to 18.58 ps; thus, the refractive index is increasing and the maximum peak is decreasing after hydrogen gas exposure, regardless of rubber type, as summarized in Table 1 and Table 2. 

The increase in the optical time delay values of rubber materials exposed to hydrogen can be attributed to hydrogen sorption, which leads to an increase in refractive index. When the same material becomes hardened because of changes in external conditions, its strength increases. Further, electromagnetic waves require more energy and time to pass through the material, resulting in an observed reduction in the peak magnitude and an extended time required for specimen penetration, consistent with published theoretical models [28].

**Figure 6:**
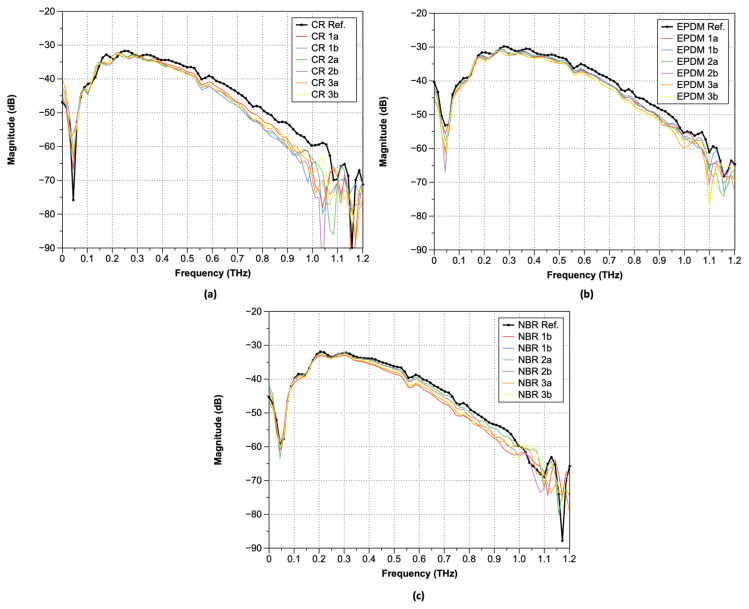
FFT spectra of the transmitted pulsed terahertz waves: (**a**) CR; (**b**) EPDM; and (**c**) NBR.

The measured terahertz time-domain electric field can be converted to the angular-frequency domain by the Fast Fourier Transform (FFT) of the time-domain signal obtained by the terahertz time-domain spectrometer. As shown in Figure 6, a comparison of the terahertz frequency-domain spectra of the three types of rubber samples (Nos. 1a–3b) shows that the waveform patterns are very similar. Under ambient conditions, water vapor peaks were observed at 0.557, 0.752, 0.988, and 1.097 THz. In the case of samples exposed to high-temperature, high-pressure hydrogen, there is a tendency for a larger variance of the magnitude over 0.8 THz, regardless of the rubber type. For the FFT results after hydrogen exposure, a phase shift occurred near 0.8 THz regardless of the type of rubbers. In terms of magnitude, CR exhibited the largest difference, followed by EPDM and NBR, with a decreasing difference. 

### 3.2. Optical Parameters of the Terahertz Waveform in the Frequency Domain

Pulsed THz-TDS is a coherence technique that measures both the amplitude and phase of pulsed terahertz waves. Coherence detection enables direct calculations of both the real ($n^{\prime }$) and imaginary parts ($n^{\prime \prime }$) of the complex refractive index (n). Herein, $n^{\prime }$ corresponds to the n of a material and is a measure of the extent to which the material slows down light. Similarly, $n^{\prime \prime }$ corresponds to the absorption coefficient (α) of a material and is a measure of the amount of light absorbed by the material. When the optical properties of a material are studied, $n\left(\omega \right)$ considers both aforementioned factors and can be expressed by Equation (1).

(1) $$n\left(\omega \right)=n^{\prime }\left(\omega \right)-{i*n}^{\prime \prime }\left(\omega \right)=c_{0}*\Delta t/d_{Sample}=(1+R)/(1-R)$$

Here, ω is the angular frequency of the terahertz wave, $c_{0}$ is the speed of light in a vacuum (2.998 × 10^8^ m/s), Δt is the time delay between the incident and transmitted terahertz pulse, and $d_{Sample}$ is the thickness of the sample. 

**Figure 7:**
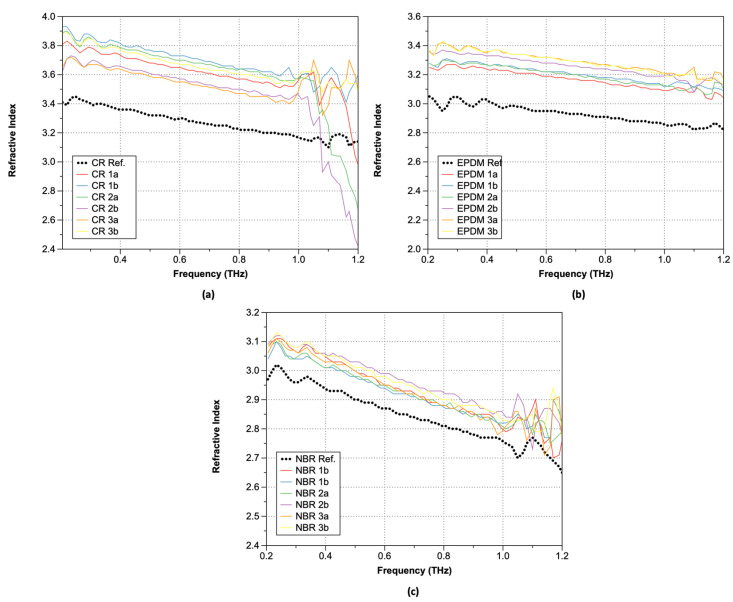
Refractive index: (**a**) CR; (**b**) EPDM; and (**c**) NBR.

Figure 7 shows the calculated refractive indexes (n) of the rubber samples after hydrogen exposure obtained using Equation (1). As shown, the exposure of rubber samples to hydrogen led to higher frequency-dependent refractive index, regardless of the rubber type. 

The complex reflectivity (R) is related to the complex transmittance (T) through the relationship R=(1−T) and is defined as the ratio of the complex amplitude of the reflected light to that of the incident light. R is expressed in terms of the magnitude and phase of the reflection coefficient. T can be calculated using Equation (2).

(2) $$T=(E_{T}/E_{i}*e^{-i\varphi })$$

Here, $E_{T}$ and $E_{i}$ are the complex amplitudes of the transmitted and incident terahertz waves, respectively, i is an imaginary unit, and φ is the phase of the transmission coefficient. The relationship between $n^{\prime \prime }$ and α in the transmitted terahertz waves can be expressed by Equation (3).

(3) $$\alpha \left(\omega \right)=2\pi fn^{\prime \prime }\left(\omega \right)=2\omega /c_{0}*n^{\prime \prime }(\omega )$$

Here, f is the frequency. Consequently, $n^{\prime \prime }$ can be expressed by Equation (4).

(4) $$n^{\prime \prime }\left(\omega \right)=c_{0}\alpha \left(\omega \right)/2\omega$$

Similarly, $n^{\prime }$ can be determined by measuring the phase shift (φ) and thickness (*d*) of the sample via Equation (5).

(5) $$n^{\prime }\left(\omega \right)=1+c_{0}*\varphi \left(\omega \right)/\omega d$$

**Figure 8:**
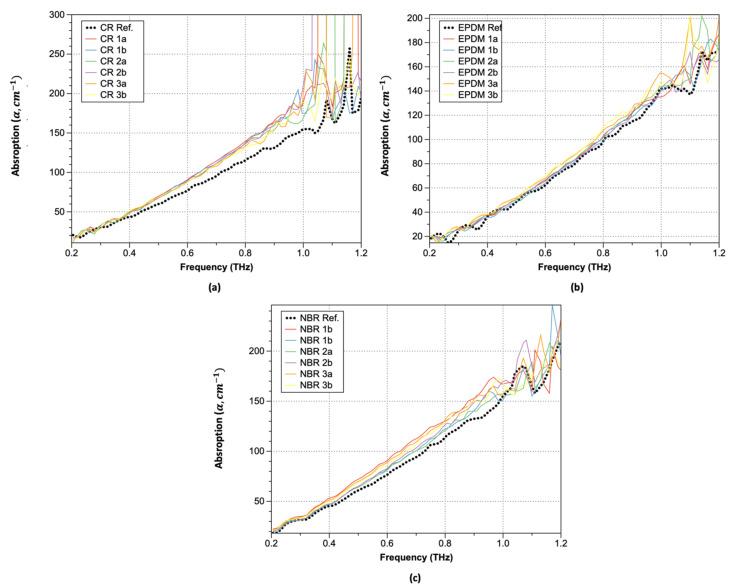
Absorption coefficients: (**a**) CR; (**b**) EPDM; and (**c**) NBR.

Plots of the obtained absorption coefficients are shown in Figure 8 (Equations (3)–(5)). As shown, the absorption coefficients change significantly at positions where the refractive index undergoes significant changes. In the lower-frequency range, similar values were observed both before and after hydrogen exposure, but the increase in the absorption coefficient of the hydrogen-exposed specimens extended up to 1.0 THz. Moreover, over 1.0 THz, the absorption coefficient exhibited a sharp increase. This trend is consistent with the optical property data analysis of hardened rubber materials. Although there are differences in the magnitude of the increase with respect to the type of rubber, the trend before and after hydrogen exposure remains consistent, and CR was the most affected by the hydrogen. In the case of CR, significant changes in the refractive index occurred after hydrogen exposure, particularly beyond 1.0 THz. For EPDM, an increase in the refractive index along with phase changes was observed after hydrogen exposure. The refractive index values also exhibited instability starting from around 1.1 THz with decreasing frequency. NBR, on the other hand, showed the smallest refractive index change, indicating that hydrogen exposure had a relatively small effect. Interestingly, NBR exhibited a phase shift opposite to that of the sample exposed to hydrogen after 1.0 THz. To analyze the characteristics before and after hydrogen exposure, we summarized the refractive index changes at a constant frequency of 0.6 THz in Table 3. After hydrogen exposure, the refractive index increased by an average of 10.12% for CR, 10.45% for NBR, and 3.14% for EPDM.

### 3.3. FTIR and Raman Spectroscopy Results

**Figure 9:**
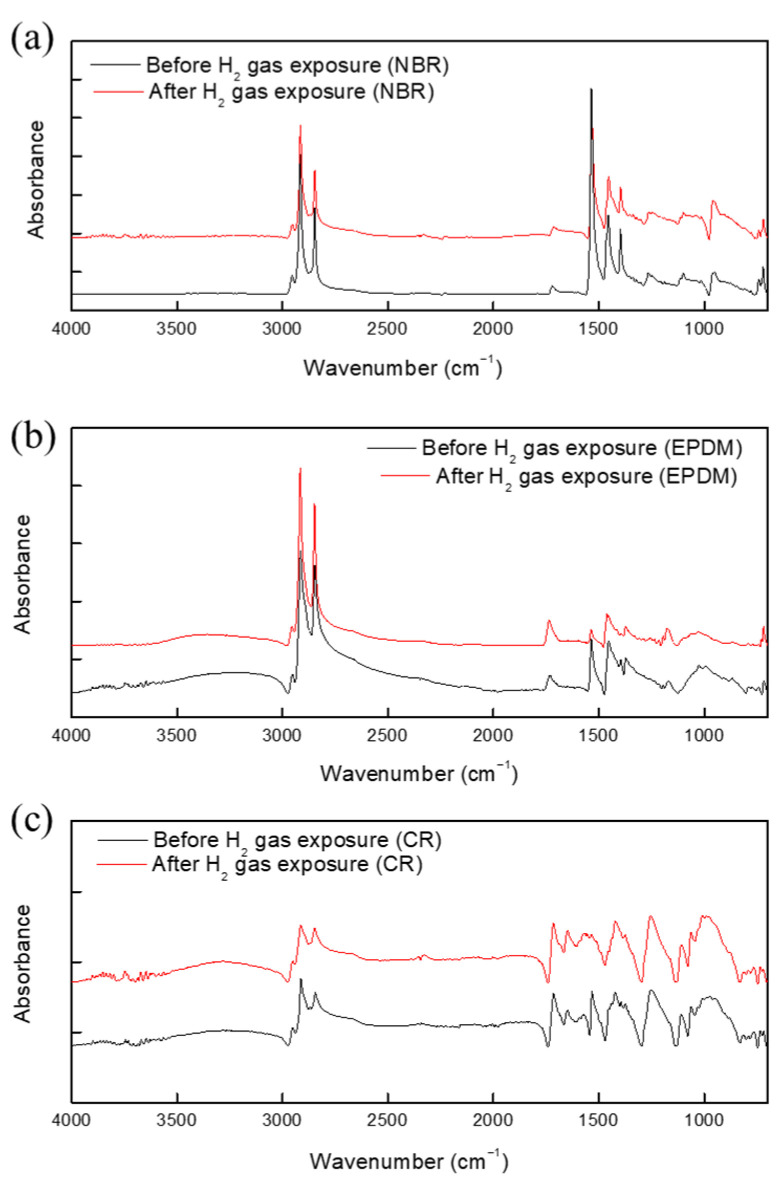
FTIR spectra of (**a**) CR, (**b**) EPDM, and (**c**) NBR before and after exposure to hydrogen gas.

Next, the specimens subjected to hydrogen gas exposure were used for FTIR and Raman spectroscopy measurements. Characteristic peaks for NBR, EPDM, and CR were observed in the FTIR spectra, as shown in Figure 9. 

**Figure 10:**
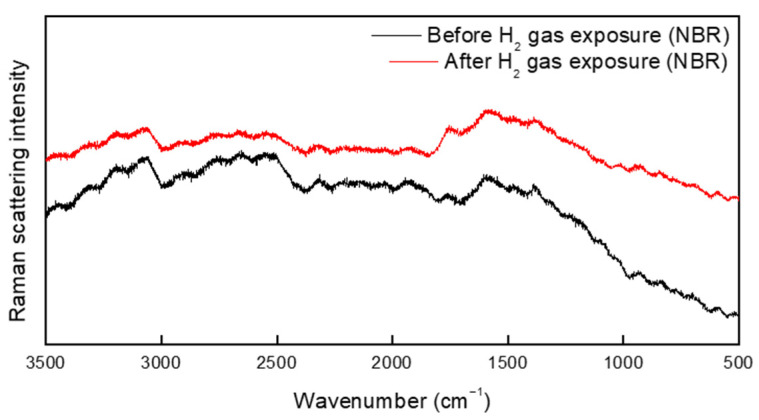
Raman spectrum of NBR before and after exposure to hydrogen gas.

**Figure 11:**
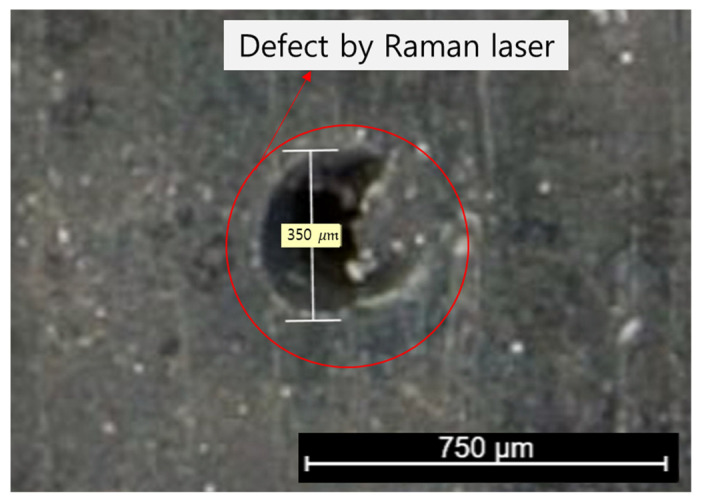
Defect induced by the Raman laser in the NBR specimen.

The Raman spectrum of NBR before and after hydrogen exposure is shown in Figure 10. No additional peaks were found in the spectra of the specimen before and after hydrogen exposure. Notably, no additional peaks were observed between 3400 and 3300 cm^−1^; this is the frequency of C=N-H vibrations arising from the hydrogenation of the cyano group (Figure 9 and Figure 10). Furthermore, the asymmetric stretching vibration of CH methylene groups at 2922 cm^−1^, symmetric stretching vibration of CH methylene groups at 2848 cm^−1^, and bending vibration of vinylene groups at 969 cm^−1^ were investigated. On the basis of these results, the hydrogenation of vinylene or cyano groups did not occur, and there was no chemical degradation induced by the breakdown of chemical bonds in the rubber molecules. As a result, exposure to high-pressure hydrogen has no effect on the chemical structure of the rubber samples. Moreover, we found no additional peaks that differed from the spectrum of the unexposed specimen. However, because the Raman spectrometer uses a laser, the rubber sample was damaged, see Figure 11. Overall, the FTIR and Raman results indicate that the terahertz system is suitable for examining changes in rubber arising from high-pressure hydrogen exposure.

## 4. Conclusions

In summary, terahertz time-domain spectroscopy is an effective method for sensing and analyzing the effects of hydrogen gas exposure on rubber. Various types of rubber including CR, EPDM, and NBR can be characterized by the analysis of the terahertz optical parameters. Crucially, the proposed method enables the rapid assessment of the quality and material properties of the final rubber product.

(1) Terahertz tests were conducted to obtain the terahertz A-scan results and optical parameters such as refractive index and absorption spectra of rubber samples (CR, EPDM, and NBR) exposed to hydrogen gas. The amplitude and optical time delay in the terahertz A-scan waveform commonly used for material property analysis can distinguish the hardness or density of the materials. All rubber samples showed a decreasing THz signal and increasing optical time delay.
(2) In the terahertz frequency spectrum as well, significant changes were observed after hydrogen exposure. Phase changes were observed from around 0.8 THz onward, and signal attenuation occurred abruptly at specific frequencies.
(3) The refractive index of CR increased by an average of 10.12%, whereas those of NBR and EPDM increased by 10.45% and 3.14%, respectively, after hydrogen exposure.
(4) The absorption coefficient spectrum exhibited similar values before and after hydrogen exposure in the lower-frequency range. However, the increase in absorption coefficient values became more pronounced up to 1.0 THz in hydrogen-exposed specimens. Furthermore, beyond 1.0 THz, the absorption coefficient values exhibited a rapid increase, followed by a decrease.

Additionally, Raman and FTIR spectroscopy measurements were performed. In the Raman spectra, sample damage was observed after testing due to the high laser power, and it was difficult to obtain meaningful results related changes after hydrogen gas exposure. In addition, the FTIR spectra did not contain new peaks arising from chemical changes in rubber caused by exposure to high-pressure hydrogen. However, limited information could be obtained because of the limited penetration depth of the light source.

We plan to carry out further verification using molecular simulations to determine the specific causes and mechanisms behind the different bands in the terahertz spectra. Crucially, the terahertz-spectroscopy-based analysis of hydrogen exposure proposed in this study is expected to be a useful non-destructive, non-contact method.

## Figures and Tables

**Table 1:** Optical time delay changes after high-pressure hydrogen gas exposure (unit: ps).

	Ref.	No. 1a	No. 1b	No. 2a	No. 2b	No. 3a	No. 3b	Avg.	∆*t*	∆*t* (%)
CR	18.00	18.57	18.73	18.67	18.43	18.47	18.60	18.58	0.58	3.21%
EPDM	17.32	17.80	17.90	17.83	17.97	18.03	18.03	17.93	0.61	3.51%
NBR	17.33	17.40	17.40	17.43	17.47	17.47	17.43	17.43	0.10	0.58%

**Table 2:** Peak value changes after high-pressure hydrogen gas exposure (unit: a.u.).

	Ref.	No. 1a	No. 1b	No. 2a	No. 2b	No. 3a	No. 3b	Avg.	∆Amp.	∆Amp. (%)
CR	0.42	0.36	0.33	0.34	0.34	0.34	0.34	0.34	−0.08	−18.13%
EPDM	0.55	0.48	0.51	0.48	0.48	0.46	0.45	0.47	−0.08	−14.50%
NBR	0.42	0.35	0.40	0.39	0.38	0.36	0.36	0.37	−0.05	−10.82%

**Table 3:** Refractive index changes after high-pressure hydrogen gas exposure.

	Ref.	No. 1a	No. 1b	No. 2a	No. 2b	No. 3a	No. 3b	Avg.	Max	Min
EPDM	2.95	3.19	3.22	3.22	3.28	3.32	3.32	3.26	3.32	3.19
CR	3.30	3.65	3.73	3.70	3.57	3.55	3.67	3.65	3.73	3.55
NBR	2.87	2.95	2.94	2.95	2.99	2.95	2.98	2.96	2.99	2.94

## Data Availability

The date presented in this study are available in the article.

## References

[1] Yamabe J., Nishimura S. (2009). Influence of fillers on hydrogen penetration properties and blister fracture of rubber composites for O-ring exposed to high-pressure hydrogen gas. Int. J. Hydrogen Energy.

[2] Yamabe J., Nishimura S. (2012). Hydrogen-induced degradation of rubber seals. Gaseous Hydrogen Embrittlement of Materials in Energy Technologies.

[3] Law V., Dowling D. Cloud Electrification as a Source of Ignition for Hydrogen Lift-Gas Airships Disasters. Proceedings of the Chaotic Modeling and Simulation International Conference.

[4] Gouran D.S., Hirokawa R.Y., Martz A.E. (1986). A critical analysis of factors related to decisional processes involved in the Challenger disaster. Cent. States Speech J..

[5] Jung J.K., Kim I.G., Kim K. (2021). Evaluation of hydrogen permeation characteristics in rubbery polymers. Curr. Appl. Phys..

[6] Yamabe J., Nishimura S. (2013). Failure behavior of rubber O-ring under cyclic exposure to high-pressure hydrogen gas. Eng. Fail. Anal..

[7] Yamabe J., Matsumoto T., Nishimura S. (2011). Application of acoustic emission method to detection of internal fracture of sealing rubber material by high-pressure hydrogen decompression. Polym. Test..

[8] Fujiwara H., Yamabe J., Nishimura S. (2012). Evaluation of the change in chemical structure of acrylonitrile butadiene rubber after high-pressure hydrogen exposure. Int. J. Hydrogen Energy.

[9] Fujiwara H., Ono H., Nishimura S. (2015). Degradation behavior of acrylonitrile butadiene rubber after cyclic high-pressure hydrogen exposure. Int. J. Hydrogen Energy.

[10] Jung J.K., Kim I.G., Chung K.S., Kim Y.-I., Kim D.H. (2021). Determination of permeation properties of hydrogen gas in sealing rubbers using thermal desorption analysis gas chromatography. Sci. Rep..

[11] Okuda R., Komatsu K., Nakamura A., Ito O., Nambu K., Saitoh H. (2019). Evaluation of released amount of hydrogen after high pressure hydrogen loading in carbonate. Results Eng..

[12] Fujiwara H. (2017). Analysis of acrylonitrile butadiene rubber (NBR) expanded with penetrated hydrogen due to high pressure hydrogen exposure. Int. Polym. Sci. Technol..

[13] Jung J.K., Kim I.G., Kim K.T., Ryu K.S., Chung K.S. (2021). Evaluation techniques of hydrogen permeation in sealing rubber materials. Polym. Test..

[14] Jeon S.K., Jung J.K., Chung N.K., Baek U.B., Nahm S.H. (2022). Investigation of Physical and Mechanical Characteristics of Rubber Materials Exposed to High-Pressure Hydrogen. Polymers.

[15] Balasooriya W., Clute C., Schrittesser B., Pinter G. (2022). A review on applicability, limitations, and improvements of polymeric materials in high-pressure hydrogen gas atmospheres. Polym. Rev..

[16] (2019). Test Methods for Evaluating Material Compatibility in Compressed Hydrogen Applications Polymers.

[17] Han D.-H. (2023). Inner defect detection of glass fiber reinforced polymer sandwich panel using pulsed terahertz imaging based on smoothing and derivative. NDT E Int..

[18] Kang L.-H., Han D.-H. (2021). Robotic-based terahertz imaging for nondestructive testing of a PVC pipe cap. NDT E Int..

[19] Freindorf M., Kraka E., Cremer D. (2012). A comprehensive analysis of hydrogen bond interactions based on local vibrational modes. Int. J. Quantum Chem..

[20] Komatsu M., Izutsu T., Ohki Y., Mizuno M., Fukunaga K., Nakamura Y., Chiwata N. Terahertz spectroscopic analysis of ethylene-propylene-diene copolymer. Proceedings of the 2014 International Symposium on Electrical Insulating Materials.

[21] Chang T., Zhang X., Cui H.-L. (2020). Thermal aging analysis of carbon black and silica filled natural rubber based on terahertz dielectric spectroscopy. Infrared Phys. Technol..

[22] Lee I.-S., Lee J.W. (2019). Effects of thermal aging on cellulose pressboard using terahertz time-domain spectroscopy. Curr. Appl. Phys..

[23] Castagnet S., Ono H., Benoit G., Fujiwara H., Nishimura S. (2017). Swelling measurement during sorption and decompression in a NBR exposed to high-pressure hydrogen. Int. J. Hydrogen Energy.

[24] Smith Z.P., Tiwari R.R., Murphy T.M., Sanders D.F., Gleason K.L., Paul D.R., Freeman B.D. (2013). Hydrogen sorption in polymers for membrane applications. Polymer.

[25] Mahankar P.S., Dhoble A.S. (2021). Review of hydraulic seal failures due to effect of medium to high temperature. Eng. Fail. Anal..

[26] Yang H.-J., Han D.-H. (2023). Study on tensile loading state sensing and estimation of opaque polymer materials using pulsed-terahertz waves. Sens. Actuators A Phys..

[27] Han D.-H., Kang L.-H. (2018). Nondestructive evaluation of GFRP composite including multi-delamination using THz spectroscopy and imaging. Compos. Struct..

[28] Peters O., Schwerdtfeger M., Wietzke S., Sostmann S., Scheunemann R., Wilk R., Holzwarth R., Koch M., Fischer B.M. (2013). Terahertz spectroscopy for rubber production testing. Polym. Test..

